# Influence of Magnesium Status on the Association of Tobacco Exposure With Depression in Old Patients With Heart Failure: A Cross‐Sectional Study of the NHANES Database

**DOI:** 10.1155/cdr/3372455

**Published:** 2026-07-19

**Authors:** Yunyu Liang, Hua Jiang, Yan Zhong, Sijie Qiu, Xinmei Li, Pingchang Xie

**Affiliations:** ^1^ Geriatrics Department, the Second Affiliated Hospital of Guangzhou University of Chinese Medicine, Guangdong Provincial Hospital of Traditional Chinese Medicine, Guangzhou, China, gdhtcm.com; ^2^ Emergency Department, the Second Affiliated Hospital of Guangzhou University of Chinese Medicine, Guangdong Provincial Key Laboratory of Research on Emergency in TCM, Guangdong Provincial Hospital of Traditional Chinese Medicine, Guangzhou, China, gdhtcm.com

**Keywords:** depression, HF, MDS, regulation effect, tobacco exposure

## Abstract

**Aim:**

The aim of this study is to examine the effect of magnesium depletion score (MDS) on the relationship between tobacco exposure and depression in older patients with heart failure (HF).

**Methods:**

Data from the National Health and Nutrition Examination Surveys (NHANES, 2005–2018) were analyzed in this cross‐sectional study. Weighted logistic regression models were used to assess associations between MDS, tobacco exposure, and depression. Propensity score matching (PSM) was applied to minimize potential confounding.

**Results:**

Among 554 eligible participants, 166 presented with depression. After covariate adjustment, quitting smoking (robustness OR = 2.179, 95% CI: 1.190–3.685), current smoking (robustness OR = 1.941, 95% CI: 0.904–3.770), and serum cotinine ≥3 ng/mL (robustness OR = 1.968, 95% CI: 0.986–3.590) were significantly associated with increased depression risk. After PSM, quitting smoking (OR = 2.16, 95% CI: 1.13–4.12) and cotinine ≥3 ng/mL (OR = 2.47, 95% CI: 1.06–5.75) remained significantly associated with depression. Compared with MDS ≤ 2 combined with no smoking, MDS ≤ 2 combined with quitting smoking (robustness OR = 2.375, 95% CI: 1.076–4.739) and MDS > 2 combined with quitting smoking (robustness OR = 3.142, 95% CI: 1.413–5.824) or currently smoking (robustness OR = 5.608, 95% CI: 1.790–13.661) were all linked to higher odds of depression. A significant multiplicative interaction between MDS and smoking was observed with *p* < 0.001. Also, MDS > 2 combined with serum cotinine ≥3 ng/mL was associated with higher odds of depression compared with MDS ≤ 2 combined with serum cotinine < 0.05 ng/mL (robustness OR = 5.258, 95% CI: 1.869–11.546).

**Conclusion:**

MDS appears a possible interaction on the association between self‐reported tobacco exposure and depression. Whether maintaining adequate magnesium status can help reduce depression risk in HF patients needs further validation.

## 1. Introduction

Heart failure (HF) is a prevalent condition among older adults, commonly attributed to underlying cardiovascular diseases (CVDs) and age‐related structural and functional cardiac changes [1]. Affecting more than 64 million people worldwide [2], HF incidence rises sharply with age, imposing a substantial disease burden [3–5]. Depression is a frequent comorbidity in HF patients, linked to elevated healthcare costs, diminished quality of life, and increased mortality risk [6]. Studies indicate that 21.5% of HF patients experience depressive symptoms and 30% experience anxiety symptoms, both substantially higher relative to the general population [7–9]. Thus, identifying HF patients at high risk for depression is essential for prevention and disease burden reduction.

Evidence increasingly highlights the role of inflammation in depression pathogenesis [10]. HF‐induced systemic and central nervous system (CNS) inflammation is a key mechanism contributing to heightened vulnerability to depression [11]. HF‐related factors such as hypoperfusion and inflammation may also cause hippocampal damage, impair neuropsychological regulation, and predispose to depressive symptoms [12].

Tobacco exposure is a modifiable environmental factor influencing both HF and depression risk in older adults, primarily through its effects on inflammation and oxidative stress [13, 14]. Cotinine, a metabolite of nicotine, serves as a reliable biomarker of tobacco smoke exposure, providing a more objective and quantitative assessment compared with self‐reported smoking status [15].

Magnesium (Mg) is an essential nutrient crucial for blood pressure regulation, myocardial metabolism, cardiac contractility, excitability, and vascular tone [16]. Emerging evidence suggests Mg plays a role in depression by influencing CNS function, modulating inflammation and oxidative stress, and regulating monoaminergic systems [17, 18]. Recent studies indicate that Mg status may interact with environmental exposures. For instance, smoking status has been shown to modify the association between Mg intake and stroke mortality [19], whereas high cotinine levels combined with low Mg intake increase asthma risk in children [20]. Additionally, sufficient Mg intake mitigates the adverse metabolic effects of pesticide and heavy metal exposure [21, 22], likely via its anti‐inflammatory and antioxidant properties.

Direct assessment of Mg status using the magnesium tolerance test (MTT) is impractical in large‐scale research. Serum Mg reflects only ~1% of body stores and does not reliably indicate total Mg status [23, 24]. Because renal Mg reabsorption is central to maintaining homeostasis [25], the magnesium depletion score (MDS) has been developed as a practical indicator of Mg status [26]. Higher MDS values have been associated with inflammation, increased HF risk in the general population [27], adverse outcomes in CVD patients, and elevated cardiovascular mortality [26, 28], making it a promising prognostic marker.

This study therefore is aimed at investigating the relationships between MDS, tobacco exposure, and depression risk in older HF patients using NHANES data, and at evaluating whether Mg status modulates the effect of tobacco exposure on depression risk.

## 2. Methods

### 2.1. Study Design and Population

This cross‐sectional study utilized data from the NHANES (2005–2018), a nationwide survey jointly conducted by the Centers for Disease Control and Prevention (CDC) and the National Center for Health Statistics (NCHS). NHANES adopts a complex, multistage, stratified probability design to ensure nationally representative sampling. Trained NCHS staff conducted household interviews, whereas physical examinations and laboratory tests were performed at mobile examination centers (MECs). Details are available at: https://wwwn.cdc.gov/nchs/nhanes/Default.aspx.

A total of 1053 HF patients aged ≥ 60 years were initially identified based on affirmative responses to the question: “Has a doctor or other health professional ever told you that you had heart failure?” [29]. Exclusion criteria included: (1) undergoing dialysis and (2) missing information on depression assessment, serum cotinine levels, MDS evaluation, or dietary intake. After applying these criteria, 554 patients remained eligible for analysis. All data were deidentified, and informed consent had been obtained by NHANES; hence, additional institutional review board approval was waived.

### 2.2. Assessment of Depression

Depressive symptoms were assessed using the Patient Health Questionnaire‐9 (PHQ‐9), administered in person at MECs. Participants rated the frequency of nine symptoms (e.g., anhedonia, low mood, sleep disturbance, fatigue, appetite changes, low self‐esteem, poor concentration, psychomotor changes, and suicidal ideation) on a 0–3 scale. A total score ≥ 10 (range 0–27) indicated clinically significant depression [30]. In addition, participants reporting antidepressant use were also categorized as having depression [31].

### 2.3. Measurement of Cotinine and MDS

According to the NHANES presentation, serum cotinine level was examined with blood samples collected in the MECs and analyzed using isotope‐diluted high performance liquid chromatography [32]. In this study, serum cotinine levels were divided into three categories, including < 0.05 ng/mL, [0.05, 3.00) ng/mL and ≥ 3.00 ng/mL [33, 34].

The MDS was calculated via aggregating the following four factors [26, 35]: (1) present diuretics use was recorded as one point, (2) current proton pump inhibitor (PPI) use was recorded as one point, (3) excessive drinking was recorded as one point, which was defined as > 1 drink/d for females and > 2 drinks/d for males, and (4) mildly decreased renal function was recorded as one point, and chronic kidney disease (CKD) was recorded as two points. According to the Chronic Kidney Disease Epidemiology Collaboration (CKD‐EPI) equation, the estimated glomerular filtration rate (eGFR) of participants was classified into three categories, in which 60 mL/(min 1.73 m^2^) ≤ eGFR < 90 mL/(min 1.73 m^2^) was defined as mildly decreased renal function, and eGFR < 60 mL/(min 1.73 m^2^) was defined as CKD [36]. The eGFR was calculated as follow: eGFR = 175 × standardized serum creatinine (Scr) − 1.154 × age − 0.203 × 1.212 (if black) × 0.742 (if female), where GFR is expressed as mL/min/1.73 m^2^ of body surface area and Scr is expressed in mg/dL. Additionally, the MDS was categorized into > 2 and ≤ 2 in this study, and a higher MDS reflected poorer Mg status.

### 2.4. Variables Selection

We also extracted variables as potential confounding factors from the database, including age, gender, race, poverty income ratio (PIR), educational level, physical activity, marital status, dyslipidemia, hypertension, diabetes mellitus (DM), cancer, stroke, coronary heart disease (CHD), antipsychotics, aldosterone receptor antagonists, angiotensin‐converting enzyme inhibitors (ACEI), course of HF, body mass index (BMI), total energy intake, the Healthy Eating Index‐2015 (HEI‐2015), as well as dietary intake levels of Mg, vitamin D, and calcium (Ca).

During the NHANES household interview, information on smoking status, the pattern of alcohol consumption, stroke, CHD, cancer, and medication was captured by self‐reported questionnaires. For smoking status, participants who responded “no” to the question “Have you smoked at least 100 cigarettes in your life?” were classified as “non‐smokers of cigarettes” (“no smoking”). The remaining participants were categorized as “former smokers of cigarettes” (“quit smoking”) or “current smokers of cigarettes” (“currently smoking”) based on their responses to the question “Do you now smoke cigarettes?” [37]. Information on physical activity was collected by the NHANES questionnaire and converted into weekly energy expenditure using the formula: weekly energy expenditure (MET · min/week) = recommended metabolic equivalent (MET) × weekly exercise time of corresponding activity (min) and were categorized with the cut − off value 450 MET · min/week expenditure (MET·min/week) = recommended metabolic equivalent (MET) × weekly exercise time of corresponding activity (min) and were categorized with the cut‐off value 450 MET.min/week.

Dietary intake information was collected through two 24 h dietary recalls; the first 24 h recall interview was conducted in person in the MEC, and the second was by telephone or mail 3 to 10 days later. The total energy intake was calculated by dietary intake plus dietary supplement. The HEI‐2015 is a diet quality index to assess adherence to the Dietary Guidelines of Americans (DGA), which focuses on the consumption of total fruits, whole fruits, total vegetables, greens and beans, whole grains, dairy foods, total protein foods, seafood and plant proteins, fatty acids, refined grains, Na, added sugars, and saturated fats. The score ranges between 0 and 100 points, and the higher score reflects the healthier eating [38, 39].

### 2.5. Statistical Analysis

Continuous variables were described using mean ± standard error (mean ± SE). T test was used for comparing characteristics between depression group and nondepression group. Categorical variables were expressed as frequency and constituent ratio (*N* [%]). Chi‐square test (*χ* ^2^) was used for the comparison. According to the NHANES recommendation, the full sample 2‐year MEC exam weight should be utilized in the analyses because we combined data from seven 2‐year cycles.

Weighted univariate logistic regression analyses were used to screen covariates associated with depression. Weighted univariate and multivariate logistic regression analyses were utilized to explore (1) the associations of MDS and tobacco exposure (including smoking and serum cotinine level) with depression, (2) the association between tobacco exposure and depression under different MDS levels, and (3) the combined effect between MDS and tobacco exposure on expression. Forest plots were drawn to reflect the prevalence situation of depression in different tobacco exposure groups under different MDS levels. The multivariate models adjusted for age, gender, race, educational level, PIR, physical activity, and antipsychotics. The 1000 bootstrap iterations have been conducted for robustness assessment. Propensity score matching (PSM) method was used to balance the distribution of covariates between the depression group and the nondepression group to reduce the impact of potential biases and confounding factors. The associations of MDS and tobacco exposure with depression as well as the influence of MDS on the relationship between tobacco exposure and depression were also assessed after PSM. The evaluation indexes were odds ratios (ORs) with 95% confidence intervals (CIs).

Two‐sided *p* < 0.05 is considered significant. Statistical analyses were performed using R (Version 4.2.0, Institute for Statistics and Mathematics, Vienna, Austria) and SAS 9.4 (SAS Institute, Cary, North Carolina, United States). Variables containing missing values (Table S1) were interpolated by multiple interpolation (detailed progress of this method was shown in Supporting Information), and sensitivity analysis was performed (Table S2).

## 3. Results

### 3.1. Characteristics of Participants

Of 1053 HF patients, 554 met eligibility criteria (Figure S1). Among them, 166 (29.96%) had depression. The mean age was 72.8 years, and 50.8% were male. Compared with the nondepression group, depressed patients had higher rates of PPI use (38.4% vs. 26.9%), higher prevalence of MDS > 2 (55.2% vs. 41.2%), and lower levels of PIR, physical activity, and antipsychotic use (all *p* < 0.05) (Table S3).

### 3.2. Associations of MDS and Tobacco Exposure With Depression in HF Patients

We first screened the covariates associated with depression in patients with HF (Table S4), the results showed that gender, physical activity, and antipsychotics were significantly linked to depression. Nevertheless, we also included some common demographic variables including age, race, educational level, and PIR. After adjusting for these covariates (Table 1), compared with HF patients who do not smoke, both who quit smoking (robustness OR = 2.179, 95% CI: 1.190–3.685) and currently smoking (robustness OR = 1.941, 95% CI: 0.904–3.770) had higher odds of depression. Also, serum cotinine ≥ 3 ng/mL was significantly associated with higher odds of depression compared with < 0.05 ng/mL (robustness OR = 1.968, 95% CI: 0.986–3.590).

**Table 1:** Associations of MDS and tobacco exposure with depression in HF patients.

Variables	Unadjusted model		Adjusted model^a^		Bootstrap
	OR (95% CI)	*p*	OR (95% CI)	*p*	OR (95% CI)
MDS					
≤ 2	Ref		Ref		Ref
> 2	1.76 (1.09–2.85)	0.022	1.64 (0.92–2.91)	0.091	1.613 (0.964–2.569)
Smoking status					
No smoking	Ref		Ref		Ref
Quit smoking	1.53 (0.92–2.56)	0.101	2.10 (1.16–3.83)	**0.016**	2.179 (1.190–3.685)
Currently smoking	1.56 (0.77–3.14)	0.211	2.12 (1.01–4.44)	**0.048**	1.941 (0.904–3.770)

Cotinine					
< 0.05	Ref		Ref		Ref
[0.05, 3)	1.22 (0.75–1.99)	0.416	1.28 (0.74–2.22)	0.375	1.125 (0.632–1.834)
≥ 3	1.73 (0.92–3.26)	0.087	2.17 (1.06–4.43)	**0.035**	1.968 (0.986–3.590)

*Note:* The bolded values in Table 1 mean *p* < 0.05 that represent statistically significant.

Abbreviations: CI, confidence interval; HF, heart failure; MDS, magnesium depletion score; OR, odds ratio; Ref, reference.

^a^Adjusted for age, gender, race, educational level, PIR, physical activity, and antipsychotics.

**Figure 1:**
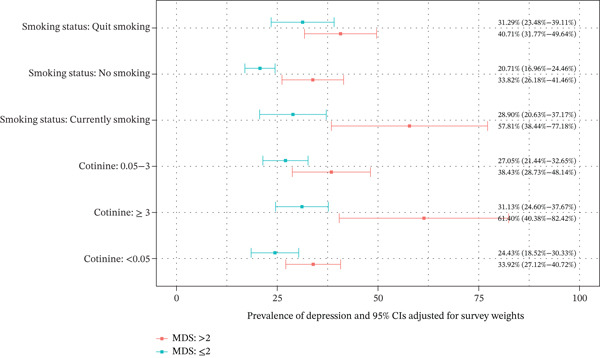
Prevalence rate of depression in HF patients with different cotinine levels and smoking status in different MDS conditions.

Then, the associations of smoking status and serum cotinine level with depression in HF patients were assessed under different MDS levels (Table S5). Among patients with MDS ≤ 2, quitting smoking and currently smoking were both linked to higher odds of depression (all *p* < 0.05). In patients with MDS > 2, quitting smoking, currently smoking, and cotinine ≥ 3 ng/mL were, respectively, associated with higher odds of depression (all *p* < 0.05). Similarly, it could be clearly observed in Figure 1 that the prevalence rate in the MDS > 2 combined with cotinine ≥ 3 ng/mL group (61.40%) or MDS > 2 combined with the currently smoking group (57.81%) was higher than other conditions, indicating that a high MDS combined with high tobacco exposure may be linked to a higher risk of depression.

In Table 2, we investigated combined effects between MDS and tobacco exposure on depression in patients with HF. After adjusting for covariates, compared with MDS ≤ 2 combined with no smoking, MDS ≤ 2 combined with quitting smoking (robustness OR = 2.375, 95% CI: 1.076–4.739) and MDS > 2 combined with quitting smoking (robustness OR = 3.142, 95% CI: 1.413–5.824) or currently smoking (robustness OR = 5.608, 95% CI: 1.790–13.661) were all linked to higher odds of depression. A significant multiplicative interaction between MDS and smoking was observed with *p* < 0.001. Also, MDS > 2 combined with serum cotinine ≥ 3 ng/mL was associated with higher odds of depression compared with MDS ≤ 2 combined with serum cotinine < 0.05 ng/mL (robustness OR = 5.258, 95% CI: 1.869–11.546).

**Table 2:** Combined effect between MDS and tobacco exposure on depression in HF patients.

Variables	Depression/total (%)	Unadjusted model		Adjusted model^a^		Bootstrap
		OR (95% CI)	*p*	OR (95% CI)	*p*	OR (95% CI)
MDS combined with smoking status						
≤ 2 and no smoking	31/128 (24.22)	Ref		Ref		Ref
≤ 2 and quit smoking	37/129 (28.68)	1.74 (0.87–3.50)	0.117	2.30 (1.10–4.82)	**0.027**	2.375 (1.076–4.739)
≤ 2 and currently smoking	15/54 (27.78)	1.56 (0.69–3.52)	0.284	1.94 (0.81–4.66)	0.303^b^	1.746 (0.599–4.321)
> 2 and no smoking	34/107 (31.78)	1.96 (1.01–3.81)	0.048	1.73 (0.80–3.75)	0.163	1.671 (0.758–3.172)
> 2 and quit smoking	38/112 (33.93)	2.63 (1.44–4.80)	0.002	3.10 (1.56–6.15)	**0.002**	3.142 (1.413–5.824)
> 2 and currently smoking	11/24 (45.83)	5.25 (1.72–16.00)	0.004	6.28 (2.09–18.87)	**0.001**	5.608 (1.790–13.661)
*p* value for interaction			< 0.001		**0.001**	

MDS combined with cotinine						
≤ 2 and < 0.05	39/164 (23.78)	Ref		Ref		Ref
≤ 2 and [0.05, 3)	20/70 (28.57)	1.15 (0.54–2.46)	0.721	1.27 (0.58–2.80)	0.541	1.094 (0.440–2.256)
≤ 2 and ≥ 3	24/77 (31.17)	1.40 (0.65–3.00)	0.384	1.59 (0.71–3.56)	0.260	1.395 (0.584–2.849)
> 2 and < 0.05	37/128 (28.91)	1.59 (0.76–3.32)	0.215	1.47 (0.65–3.37)	0.352	1.436 (0.710–2.585)
> 2 and [0.05, 3)	30/78 (38.46)	1.93 (0.92–4.07)	0.082	1.76 (0.79–3.92)	0.165	1.532 (0.704–2.999)
> 2 and ≥ 3	16/37 (43.24)	4.92 (1.78–13.60)	0.003	5.77 (2.22–14.99)	**< 0.001**	5.258 (1.869–11.546)
*p* value for interaction			0.142		0.078	

*Note:* The bolded values in Table 2 mean *p* < 0.05 that represent statistically significant.

Abbreviations: CI, confidence interval; HF, heart failure; MDS, magnesium depletion score; OR, odds ratio; Ref, reference.

^a^Adjusted for age, gender, race, educational level, PIR, physical activity, and antipsychotics.

^b^Fisher′s exact test.

### 3.3. Potential Regulating Effect of MDS on Association Between Tobacco Exposure and Depression After PSM

After PSM, baseline characteristics between depression and nondepression groups were balanced (Table S6). Quitting smoking (OR = 2.16, 95% CI: 1.13–4.12) and cotinine ≥ 3 ng/mL (OR = 2.47, 95% CI: 1.06–5.75) remained significantly associated with depression (Table 3). Among patients with MDS > 2, quitting smoking, current smoking, and cotinine ≥ 3 ng/mL were each linked to higher depression risk (all *p* < 0.05) (Table S7).

**Table 3:** Associations of MDS and tobacco exposure with depression after PSM.

Variables	OR (95% CI)	*p*
MDS		
≤ 2	Ref	
> 2	1.28 (0.72–2.28)	0.402

Smoking status		
No smoking	Ref	
Quit smoking	2.16 (1.13–4.12)	**0.021**
Currently smoking	1.88 (0.77–4.58)	0.161

Cotinine		
< 0.05	Ref	
[0.05, 3)	1.33 (0.83–2.13)	0.225
≥ 3	2.47 (1.06–5.75)	**0.037**

*Note:* The bolded values in Table 3 mean *p* < 0.05 that represent statistically significant.

Abbreviations: CI, confidence interval; MDS, magnesium depletion score; OR, odds ratio; PSM, propensity score matching; Ref, reference.

### 3.4. Associations of MDS and Tobacco Exposure With Depression (Leaving Out Antidepressant Use)

In addition, we explored the associations of MDS and tobacco exposure with depression, where the diagnosis of depression was based only on depression symptoms instead of being combined with antidepressant use, to reduce the influence from inversion of cause and outcome. Table S8 and S9, respectively, showed the characteristics of participants and the covariates screening. After adjusting for age, gender, race, educational level, PIR, marital status, hypertension, CHD, and antidepressants, we found that among HF patients with MDS > 2, currently smoking (OR = 4.44, 95% CI: 1.13–17.38) and serum cotinine ≥ 3 ng/mL (OR = 3.50, 95% CI: 1.52–8.09) were both associated with higher odds of depression (Table 4).

**Table 4:** Associations of MDS and tobacco exposure with depression (leaving out antidepressant use).

Variables	Unadjusted model		Adjusted model^a^	
	OR (95% CI)	*p*	OR (95% CI)	*p*
MDS: ≤ 2 (*n* = 311)				
Smoking status				
No smoking	Ref		Ref	
Quit smoking	1.69 (0.75–3.83)	0.200	1.79 (0.84–3.80)	0.128
Currently smoking	1.34 (0.49–3.68)	0.560	0.76 (0.28–2.11)	0.597

MDS: > 2 (*n* = 243)				
Smoking status				
No smoking	Ref		Ref	
Quit smoking	1.87 (0.69–5.09)	0.214	2.74 (0.93–8.07)	0.066
Currently smoking	2.99 (0.93–9.59)	0.064	4.44 (1.13–17.38)	**0.033**

MDS: ≤ 2 (*n* = 311)				
Cotinine				
< 0.05	Ref		Ref	
[0.05, 3)	1.23 (0.58–2.62)	0.587	1.25 (0.56–2.75)	0.579
≥ 3	1.01 (0.36–2.83)	0.981	0.43 (0.13–1.44)	0.166

MDS: > 2 (*n* = 243)				
Cotinine				
< 0.05	Ref		Ref	
[0.05, 3)	1.69 (0.63–4.49)	0.287	1.74 (0.59–5.14)	0.310
≥ 3	2.32 (0.88–6.09)	0.086	3.50 (1.52–8.09)	**0.004**

*Note:* The bolded values in Table 4 mean *p* < 0.05 that represent statistically significant.

Abbreviations: CI, confidence interval; MDS, magnesium depletion score; OR, odds ratio; PSM, propensity score matching; Ref, reference.

^a^Adjusted for age, gender, race, educational level, PIR, marital status, hypertension, CHD, and antidepressants.

## 4. Discussion

This study demonstrated that tobacco exposure—whether assessed by smoking status or serum cotinine—was significantly associated with depression among older HF patients. Importantly, Mg status, reflected by MDS, appeared to have an interaction on this relationship.

To the best of our knowledge, it was the first to discuss the potential effect of Mg status on the association between tobacco exposure and depression risk in patients with HF. Epidemiological evidence has shown that smoking might cause depression [40]. Tobacco smoke contains about 4000 chemical substances, many of which are toxicants, especially nicotine and metals [41, 42]. Metal ions, such as aluminum, manganese, zinc, and iron, have been reported to be linked to anxiety, depression, and insomnia symptoms. The mechanisms involved are related to nerve cell apoptosis and necroptosis, neurotoxicity, and the regulation of mitochondrial function and energy production [43–45]. In the human body, Mg is connected with brain biochemistry and the fluidity of the neuronal membrane. Also, Mg preparations are perceived as effective agents in the treatment of asthma, heart diseases, arrhythmias, and so on [46]. According to these theoretical bases, it was speculated that tobacco smoke exposure, as one of the most common and controllable environmental factors, plays an important role in the occurrence and development of depression and may interact with Mg status in the human body. Results in the present study showed the significantly positive associations of quitting/currently smoking and serum cotinine levels with depression odds in HF patients, and Mg status has a possible interaction on these relationships, which relatively supplemented the influence of Mg on the association between tobacco exposure and the depression literature blank in the HF population.

In the current study, Mg status was assessed using MDS, which is a burgeoning index for Mg status evaluation developed by Fan et al. [26]. Serum Mg may not accurately reflect the overall Mg status due to kidney absorbing more than 80% of plasma Mg for maintaining Mg homeostasis. Differently, the MDS aggregates four established risk factors and considers the pathophysiological factors influencing the kidneys′ reabsorption capability, where a higher MDS indicates a more severe state of Mg deficiency. The MDS scales take both gender and PPI into account, which were significantly different between nondepression group and depression group among our participants. Heart and brain share common risk factors, such as hypertension, DM, and smoking, and are similarly affected by systemic inflammation, atherosclerosis, and dysfunction of the neuroendocrine system [47, 48]. In fact, sex‐related differences develop and modify the heart‐brain axis during the entire lifespan [49]. Clinical data suggested a significant sex difference in inflammatory and innate immune responses, with females showing higher baseline levels of circulating inflammatory markers and more pronounced production of proinflammatory cytokines in response to different injuries [50, 51]. Herein, in the multivariate analyses, we have included gender as a covariate in the adjustment. Diuretics can promote Mg excretion, and PPI influences intestinal absorption of Mg [52]. Ye et al. [28] explored the association between individual items of MDS and cardiovascular outcomes and found that diuretic use and renal dysfunction were independently associated with poor outcomes. Although diuretics are recommended for patients with HF, it should be focused that even mild declines in renal function could cause increased risk of CVD and mortality.

Our results showed that MDS ≤ 2 had a potential interaction on self‐reported tobacco exposure related depression in HF patients both before and after PSM. Cotinine is a significant proximal metabolite of nicotine and is regarded as a trustworthy and sensitive indicator of tobacco smoke exposure within the past 72 h [33, 53], and therefore, cotinine has been recognized as a distinctive chemical reflecting an individual′s degree of tobacco smoke exposure in recent years. Besides, plasma cotinine can accurately discriminate between active and passive smoking and quantifies cigarette smoke exposure [54]. In addition to cotinine levels, to evaluate the long‐term effects of tobacco smoke exposure, we also included smoking status self‐reported by participants during the NHANES face‐to‐face interview. After adjusting for covariates, among HF patients with low MDS levels, quitting smoking, currently smoking and serum cotinine ≥ 3 ng/mL was associated with higher odds of depression. These findings indicated that a healthy Mg status may be crucial in reducing depression risk among HF patients with both short‐term and long‐term tobacco exposure, even a previous exposure. Due to HF patients with antipsychotics use in the depression group being significantly more than those in the nondepression group, we investigated the potential interaction of MDS on the relationship between tobacco exposure and depression that evaluated leaving out antidepressant use. Similarly, the results showed that there was a possible interaction of MDS on tobacco exposure related depression that was significant in the self‐reported currently smoking group. Notably, among the components of MDS, PPI use may serve as a proxy for underlying comorbidities or clinical conditions, which could introduce residual confounding into the observed association.

This study first explored the influence of Mg status reflected by MDS on the association between tobacco exposure and depression in patients with HF, which may provide some references for risk stratification and prevention intervention in HF patients at an old age. Also, the positive effects of adequate Mg status on depression in different tobacco exposure dimensions were validated through multiple sensitivity analyses, and therefore, the results were relatively robust and reliable. However, there are still some limitations in this study. The causal associations of Mg status and tobacco smoke exposure with depression in HF could not be concluded due to the cross‐sectional design and no information was recorded in the NHANES database on chronological order. Diagnosis of depression was through a questionnaire during the MEC interview, which may cause recalling bias. Disease attributes of HF were unavailable in the NHANES database, which may cause bias. In addition, since the effect of Mg deficiency may be conflated with the effect of disease severity, a further prospective cohort study focusing on the potential regulating effect of Mg status on the association between tobacco smoke exposure and depression in HF patients is required.

## 5. Conclusion

Adequate Mg status, as reflected by a lower MDS, was associated with reduced risk of tobacco exposure–related depression among older HF patients. These findings highlight the importance of monitoring and optimizing Mg status in clinical management, which may represent a feasible strategy for mitigating depression risk in this vulnerable population.

## Author Contributions

Y.L. designed the study and drafted the manuscript. H.J., Y.Z., and S.Q. investigated, collected, analyzed, and interpreted data. X.L. and P.X. critically reviewed, edited, and approved the manuscript. Y.L. and H.J. contributed equally to the work.

## Funding

A Randomized Controlled Clinical Trial on the Treatment of Stable Chronic Heart Failure in the Elderly with Formula Granules vs. Traditional Decoction Pieces of Academician Chen Keji’s Empirical Prescription “Xinli Formula”supported by the Guangdong Provincial Basic and Applied Basic Research Foundation (Project No. 2022A1515220016).

## Disclosure

All authors read and approved of the final manuscript.

## Ethics Statement

Due to the data from included NHANES databases being publicly available, ethical approval had been waived from the IRB of the Second Affiliated Hospital of Guangzhou University of Chinese Medicine.

## Consent

All authors agree to publish this article.

## Conflicts of Interest

The authors declare no conflicts of interest.

## Supporting Information

Additional supporting information can be found online in the Supporting Information section.

## Supporting information


**Supporting Information 1** File S1: The method for missing values process.

**Supporting Information 2** File S2: Tables S1–8.

**Supporting Information 3** Figure S1: Flowchart of the study process.

## Data Availability

The datasets generated during and/or analyzed during the current study are available in the NHANES database, https://wwwn.cdc.gov/nchs/nhanes/.
