# Supplementary material for: Influence of Magnesium Status on the Association of Tobacco Exposure With Depression in Old Patients With Heart Failure: A Cross‐Sectional Study of the NHANES Database
Source: Cardiovasc Ther. 2026 Jul 19;2026:3372455. doi: 10.1155/cdr/3372455 (PMC13382352; doi:10.1155/cdr/3372455)
Supplement: Supplementary file 1 — Supporting Information 1 File S1: The method for missing values process. [file CDR-2026-3372455-s001.docx]

This study employed the multiple imputation (MI) method to handle the missing values in the dataset (final_data). The specific parameters and steps are as follows:

1. Key settings of interpolation: Set the random seed to 12345678 to ensure the reproducibility of the results. A total of 5 interpolation data sets were generated, and the interpolation results are rounded to 1 decimal place (round=1).

2. Variables progress: The CAD was set as a categorical variable and included in the imputation model; the variables to be imputed included DF, VD, PIR, CHD, physical activity, and BMI.

3. Interpolation algorithm: The fully conditioned regularized parametric multivariate regression method (FCS RegPMM) was adopted for interpolation. This method is suitable for filling in missing values for mixed-type variables (categorical + continuous) and can better adapt to the characteristics of the dataset in this study where categorical variables and continuous variables coexist.
