# Supplementary material for: Influence of Magnesium Status on the Association of Tobacco Exposure With Depression in Old Patients With Heart Failure: A Cross‐Sectional Study of the NHANES Database
Source: Cardiovasc Ther. 2026 Jul 19;2026:3372455. doi: 10.1155/cdr/3372455 (PMC13382352; doi:10.1155/cdr/3372455)
Supplement: Supplementary file 2 — Supporting Information 2 File S2: Tables S1–8. [file CDR-2026-3372455-s002.docx]

**Table S1. Variables containing missing values**

| Variables | N (%) |
| --- | --- |
| PIR | 38 (6.86) |
| CHD | 18 (3.25) |
| Course of HF | 57 (10.29) |
| BMI | 14 (2.53) |
| Vitamin D | 5 (0.90) |
| Physical activity | 42 (7.58) |

PIR: poverty income ratio, CHD: coronary heart disease, HF: heart failure, BMI: body mass index.

**Table S2. Characteristics of participants before and after interpolation**

| Variables | After interpolation | Before interpolation | Statistics | *P* |
| --- | --- | --- | --- | --- |
| Course of HF, Mean (SE) | 10.28 (0.50) | 10.20 (0.55) | t=0.58 | 0.563 |
| Vitamin D, Mean (SE) | 35.72 (0.77) | 35.76 (0.76) | t=-0.49 | 0.627 |
| PIR, Mean (SE) | 2.47 (0.08) | 2.50 (0.08) | t=-1.52 | 0.131 |
| CHD, n (%) |  |  | χ^2^=0.011 | 0.918 |
| No | 314 (56.38) | 303 (56.35) |  |  |
| Yes | 240 (43.62) | 233 (43.65) |  |  |
| BMI (kg/m^2^), n (%) | 31.57 (0.35) | 31.54 (0.36) | t=0.56 | 0.580 |
| Physical activity (MET·min/week), Mean (SE) | 412.96 (40.16) | 392.72 (37.76) | t=1.57 | 0.119 |

PIR: poverty income ratio, CHD: coronary heart disease, HF: heart failure, BMI: body mass index.

**Table S3. Comparation of characteristics of HF patients between non-depression group and depression group**

| Variables | Total (n=554) | Depression | | Statistics | *P* |
| --- | --- | --- | --- | --- | --- |
|  |  | No (n=388) | Yes (n=166) |  |  |
| Age, years, Mean (SE) | 72.82 (0.33) | 72.96 (0.42) | 72.54 (0.49) | t=0.63 | 0.528 |
| Gender, n (%) |  |  |  | χ^2^=8.176 | **0.004** |
| Male | 303 (50.81) | 232 (56.31) | 71 (39.15) |  |  |
| Female | 251 (49.19) | 156 (43.69) | 95 (60.85) |  |  |
| Race, n (%) |  |  |  | χ^2^=0.947 | 0.623 |
| White | 321 (79.96) | 222 (79.60) | 99 (80.71) |  |  |
| Black | 116 (9.93) | 87 (10.65) | 29 (8.39) |  |  |
| Others | 117 (10.12) | 79 (9.75) | 38 (10.90) |  |  |
| PIR | 2.47 (0.08) | 2.54 (0.08) | 2.33 (0.18) | t=1.07 | 0.287 |
| PIR, n (%) |  |  |  | χ^2^=3.922 | **0.048** |
| <1.3 | 182 (23.47) | 112 (20.26) | 70 (30.30) |  |  |
| ≥1.3 | 372 (76.53) | 276 (79.74) | 96 (69.70) |  |  |
| Educational level, n (%) |  |  |  | χ^2^=2.054 | 0.358 |
| Under high school | 160 (22.52) | 103 (20.33) | 57 (27.17) |  |  |
| High school | 161 (30.70) | 114 (31.80) | 47 (28.37) |  |  |
| Above high school | 233 (46.78) | 171 (47.88) | 62 (44.46) |  |  |
| Physical activity, MET·min/week, Mean (SE) | 412.96 (40.16) | 517.77 (58.65) | 190.62 (31.35) | t=4.43 | **<0.001** |
| Physical activity, MET·min/week, n (%) |  |  |  | χ^2^=17.870 | **<0.001** |
| <450 | 430 (77.43) | 286 (71.54) | 144 (89.94) |  |  |
| ≥450 | 124 (22.57) | 102 (28.46) | 22 (10.06) |  |  |
| Marital status, n (%) |  |  |  | χ^2^=2.205 | 0.332 |
| Single | 25 (3.71) | 17 (2.77) | 8 (5.69) |  |  |
| Married | 267 (54.18) | 194 (56.11) | 73 (50.09) |  |  |
| Unknown | 262 (42.11) | 177 (41.12) | 85 (44.22) |  |  |
| Dyslipidemia, n (%) |  |  |  | χ^2^=0.873 | 0.350 |
| No | 45 (7.13) | 36 (7.97) | 9 (5.35) |  |  |
| Yes | 509 (92.87) | 352 (92.03) | 157 (94.65) |  |  |
| Hypertension, n (%) |  |  |  | χ^2^=1.978 | 0.160 |
| No | 16 (2.03) | 9 (1.36) | 7 (3.46) |  |  |
| Yes | 538 (97.97) | 379 (98.64) | 159 (96.54) |  |  |
| DM, n (%) |  |  |  | χ^2^=0.386 | 0.535 |
| No | 273 (53.46) | 191 (52.23) | 82 (56.08) |  |  |
| Yes | 281 (46.54) | 197 (47.77) | 84 (43.92) |  |  |
| Cancer, n (%) |  |  |  | χ^2^=1.117 | 0.291 |
| No | 422 (70.49) | 291 (68.73) | 131 (74.22) |  |  |
| Yes | 132 (29.51) | 97 (31.27) | 35 (25.78) |  |  |
| Stroke, n (%) |  |  |  | χ^2^=3.189 | 0.074 |
| No | 447 (80.80) | 321 (83.39) | 126 (75.31) |  |  |
| Yes | 107 (19.20) | 67 (16.61) | 40 (24.69) |  |  |
| CHD, n (%) |  |  |  | χ^2^=2.918 | 0.088 |
| No | 314 (56.38) | 230 (59.78) | 84 (49.16) |  |  |
| Yes | 240 (43.62) | 158 (40.22) | 82 (50.84) |  |  |
| Antipsychotics, n (%) |  |  |  | χ^2^=6.533 | **0.011** |
| No | 545 (98.90) | 385 (99.63) | 160 (97.35) |  |  |
| Yes | 9 (1.10) | 3 (0.37) | 6 (2.65) |  |  |
| Aldosterone receptor antagonists, n (%) |  |  |  | χ^2^=0.415 | 0.520 |
| No | 495 (89.98) | 344 (89.22) | 151 (91.59) |  |  |
| Yes | 59 (10.02) | 44 (10.78) | 15 (8.41) |  |  |
| ACEI, n (%) |  |  |  | χ^2^=0.815 | 0.367 |
| No | 534 (95.60) | 375 (96.52) | 159 (93.64) |  |  |
| Yes | 20 (4.40) | 13 (3.48) | 7 (6.36) |  |  |
| Course of HF, Mean (SE) | 10.28 (0.50) | 10.22 (0.59) | 10.43 (0.81) | t=-0.21 | 0.832 |
| BMI, kg/m^2^, Mean (SE) | 31.57 (0.35) | 31.23 (0.39) | 32.28 (0.68) | t=-1.30 | 0.196 |
| BMI, kg/m^2^, n (%) |  |  |  | χ^2^=2.581 | 0.275 |
| <25 | 92 (15.57) | 69 (16.34) | 23 (13.96) |  |  |
| [25, 30) | 170 (30.01) | 120 (31.98) | 50 (25.83) |  |  |
| ≥30 | 292 (54.42) | 199 (51.69) | 93 (60.21) |  |  |
| Vitamin D | 35.72 (0.77) | 36.04 (0.96) | 35.04 (1.37) | t=0.57 | 0.571 |
| Total energy intake, Mean (SE) | 1757.48 (40.45) | 1758.37 (48.70) | 1755.59 (70.31) | t=0.03 | 0.974 |
| HEI-2015, Mean (SE) | 52.66 (0.77) | 53.52 (0.88) | 50.82 (1.29) | t=1.65 | 0.101 |
| Mg, mg, Mean (SE) | 252.37 (5.76) | 257.85 (7.48) | 240.75 (8.78) | t=1.40 | 0.166 |
| Ca, mg, Mean (SE) | 805.01 (24.30) | 820.92 (32.56) | 771.28 (40.47) | t=0.86 | 0.391 |
| Alcohol, gm, Mean (SE) | 6.74 (0.84) | 6.53 (1.18) | 7.17 (1.38) | t=-0.32 | 0.750 |
| eGFR, Mean (SE) | 66.00 (1.12) | 66.14 (1.20) | 65.70 (2.33) | t=0.17 | 0.867 |
| Score of eGFR, n (%) |  |  |  | χ^2^=0.990 | 0.610 |
| 0 | 67 (11.60) | 50 (12.93) | 17 (8.77) |  |  |
| 1 | 267 (48.40) | 182 (47.66) | 85 (49.96) |  |  |
| 2 | 220 (40.00) | 156 (39.41) | 64 (41.27) |  |  |
| Diuretics, n (%) |  |  |  | χ^2^=0.775 | 0.379 |
| No | 220 (36.96) | 151 (38.45) | 69 (33.79) |  |  |
| Yes | 334 (63.04) | 237 (61.55) | 97 (66.21) |  |  |
| PPI, n (%) |  |  |  | χ^2^=4.125 | **0.042** |
| No | 398 (69.40) | 292 (73.06) | 106 (61.62) |  |  |
| Yes | 156 (30.60) | 96 (26.94) | 60 (38.38) |  |  |
| Score of drinking, n (%) |  |  |  | χ^2^=0.094 | 0.759 |
| 0 | 474 (84.79) | 336 (84.35) | 138 (85.71) |  |  |
| 1 | 80 (15.21) | 52 (15.65) | 28 (14.29) |  |  |
| MDS, n (%) |  |  |  | χ^2^=5.717 | **0.017** |
| ≤2 | 311 (54.34) | 228 (58.83) | 83 (44.80) |  |  |
| >2 | 243 (45.66) | 160 (41.17) | 83 (55.20) |  |  |
| Score of MDS, n (%) |  |  |  | χ^2^=11.270 | **0.046** |
| 0 | 20 (2.12) | 12 (2.04) | 8 (2.30) |  |  |
| 1 | 107 (17.02) | 78 (17.31) | 29 (16.40) |  |  |
| 2 | 184 (35.19) | 138 (39.48) | 46 (26.10) |  |  |
| 3 | 173 (32.97) | 116 (30.39) | 57 (38.44) |  |  |
| 4 | 67 (12.52) | 43 (10.72) | 24 (16.33) |  |  |
| 5 | 3 (0.18) | 1 (0.06) | 2 (0.42) |  |  |
| Smoking status, n (%) |  |  |  | χ^2^=3.527 | 0.171 |
| No smoking | 235 (43.41) | 170 (46.74) | 65 (36.34) |  |  |
| Quit smoking | 241 (45.54) | 166 (42.91) | 75 (51.13) |  |  |
| Currently smoking | 78 (11.05) | 52 (10.35) | 26 (12.54) |  |  |
| Cotinine, ng/mL, Mean (SE) | 40.59 (5.23) | 37.53 (6.83) | 47.08 (7.15) | t=-0.74 | 0.461 |
| Cotinine, ng/mL, n (%) |  |  |  | χ^2^=3.822 | 0.148 |
| <0.05 | 292 (58.96) | 216 (61.74) | 76 (53.06) |  |  |
| [0.05, 3) | 148 (23.21) | 98 (22.85) | 50 (24.00) |  |  |
| ≥3 | 114 (17.82) | 74 (15.41) | 40 (22.95) |  |  |

t: t test, χ^2^: chi-square test.

HF: heart failure, SE: standard error, PIR: poverty income ratio, DM: diabetes mellitus, CHD: coronary heart disease, ACEI: angiotensin-converting enzyme inhibitors, BMI: body mass index, HEI-2015: the Healthy Eating Index-2015, Mg: magnesium, Ca: calcium, eGFR: estimated glomerular filtration rate, PPI: proton pump inhibitor, MDS: magnesium depletion score.

**Table S4. Covariates associated with depression**

| Variables | OR (95% CI) | *P* |
| --- | --- | --- |
| Age | 0.99 (0.96-1.02) | 0.516 |
| Gender |  |  |
| Male | Ref |  |
| Female | 2.00 (1.23-3.27) | **0.006** |
| Race |  |  |
| White | Ref |  |
| Black | 0.78 (0.45-1.33) | 0.349 |
| Others | 1.10 (0.60-2.04) | 0.751 |
| PIR | 0.90 (0.73-1.10) | 0.305 |
| PIR |  |  |
| <1.3 | Ref |  |
| ≥1.3 | 0.58 (0.34-1.01) | 0.054 |
| Educational level |  |  |
| Under high school | Ref |  |
| High school | 0.67 (0.37-1.20) | 0.173 |
| Above high school | 0.69 (0.38-1.27) | 0.233 |
| Physical activity | 0.99 (0.99-0.99) | **0.002** |
| Physical activity level |  |  |
| <450 | Ref |  |
| ≥450 | 0.28 (0.15-0.53) | **<0.001** |
| Marital status |  |  |
| Single | Ref |  |
| Married | 0.44 (0.11-1.69) | 0.227 |
| Unknown | 0.52 (0.13-2.04) | 0.348 |
| Dyslipidemia |  |  |
| No | Ref |  |
| Yes | 1.53 (0.61-3.81) | 0.356 |
| Hypertension |  |  |
| No | Ref |  |
| Yes | 0.38 (0.09-1.59) | 0.183 |
| DM |  |  |
| No | Ref |  |
| Yes | 0.86 (0.52-1.41) | 0.538 |
| Cancer |  |  |
| No | Ref |  |
| Yes | 0.76 (0.46-1.27) | 0.294 |
| Stroke |  |  |
| No | Ref |  |
| Yes | 1.65 (0.94-2.88) | 0.080 |
| CHD |  |  |
| No | Ref |  |
| Yes | 1.54 (0.93-2.54) | 0.094 |
| Antipsychotics |  |  |
| No | Ref |  |
| Yes | 7.34 (1.20-44.93) | **0.032** |
| Aldosterone receptor antagonists |  |  |
| No | Ref |  |
| Yes | 0.76 (0.32-1.79) | 0.524 |
| ACEI |  |  |
| No | Ref |  |
| Yes | 1.88 (0.45-7.83) | 0.380 |
| Course of HF | 1.00 (0.98-1.02) | 0.825 |
| BMI | 1.02 (0.99-1.05) | 0.187 |
| BMI |  |  |
| <25 | Ref |  |
| [25, 30) | 0.95 (0.46-1.94) | 0.876 |
| ≥30 | 1.36 (0.69-2.71) | 0.373 |
| Vitamin D | 1.00 (0.99-1.01) | 0.559 |
| Total energy intake | 1.00 (1.00-1.00) | 0.974 |
| HEI-2015 | 0.99 (0.97-1.00) | 0.077 |
| Mg | 1.00 (1.00-1.00) | 0.153 |
| Ca | 1.00 (1.00-1.00) | 0.385 |
| Alcohol | 1.00 (0.99-1.01) | 0.736 |

OR: odds ratio, CI: confidence interval, Ref: reference, PIR: poverty income ratio, DM: diabetes mellitus, CHD: coronary heart disease, ACEI: angiotensin-converting enzyme inhibitors, HF: heart failure, BMI: body mass index, HEI-2015: the Healthy Eating Index-2015, Mg: magnesium, Ca: calcium.

**Table S5. Association between tobacco exposure and depression under different MDS levels**

| Variables | Unadjusted model | | Adjusted model* | |
| --- | --- | --- | --- | --- |
|  | OR (95% CI) | *P* | OR (95% CI) | *P* |
| MDS: ≤2 (n=311) |  |  |  |  |
| Smoking status |  |  |  |  |
| No smoking | Ref |  | Ref |  |
| Quit smoking | 1.74 (0.92-3.30) | 0.087 | 2.45 (1.25-4.79) | **0.010** |
| Currently smoking | 1.56 (0.78-3.10) | 0.204 | 2.22 (1.03-4.74) | **0.041** |
| MDS: >2 (n=243) |  |  |  |  |
| Smoking status |  |  |  |  |
| No smoking | Ref |  | Ref |  |
| Quit smoking | 1.34 (0.72-2.51) | 0.345 | 1.83 (1.02-3.29) | **0.042** |
| Currently smoking | 2.68 (1.24-5.79) | 0.014 | 3.39 (1.62-7.09) | **0.002** |
| MDS: ≤2 (n=311) |  |  |  |  |
| Cotinine |  |  |  |  |
| <0.05 | Ref |  | Ref |  |
| [0.05, 3) | 1.15 (0.58-2.25) | 0.685 | 1.34 (0.65-2.75) | 0.422 |
| ≥3 | 1.40 (0.69-2.85) | 0.349 | 1.73 (0.76-3.92) | 0.183 |
| MDS: >2 (n=243) |  |  |  |  |
| Cotinine |  |  |  |  |
| <0.05 | Ref |  | Ref |  |
| [0.05, 3) | 1.22 (0.68-2.17) | 0.496 | 1.22 (0.59-2.53) | 0.579 |
| ≥3 | 3.10 (1.45-6.62) | 0.005 | 3.77 (1.75-8.14) | **0.001** |

MDS: magnesium depletion score, OR: odds ratio, CI: confidence interval, Ref: reference.

*Adjusted for age, gender, race, educational level, PIR, physical activity and antipsychotics.

**Table S6. Characteristics of participants after balancing the distribution of covariates by PSM method**

| Variables | Depression | | Statistics | *P* |
| --- | --- | --- | --- | --- |
|  | No (n=142) | Yes (n=142) |  |  |
| Age, years, Mean (SE) | 73.15 (0.64) | 72.51 (0.45) | t=0.73 | 0.468 |
| Gender, n (%) |  |  | χ^2^=0.096 | 0.757 |
| Male | 65 (38.93) | 61 (41.21) |  |  |
| Female | 77 (61.07) | 81 (58.79) |  |  |
| Race, n (%) |  |  | χ^2^=3.128 | 0.209 |
| White | 78 (78.32) | 88 (81.98) |  |  |
| Black | 31 (11.22) | 23 (7.09) |  |  |
| Others | 33 (10.46) | 31 (10.93) |  |  |
| PIR | 2.19 (0.16) | 2.36 (0.16) | t=-0.66 | 0.513 |
| PIR, n (%) |  |  | χ^2^=0.105 | 0.746 |
| <1.3 | 57 (29.90) | 56 (27.82) |  |  |
| ≥1.3 | 85 (70.10) | 86 (72.18) |  |  |
| Educational level, n (%) |  |  | χ^2^=0.011 | 0.995 |
| Under high school | 48 (26.52) | 46 (26.13) |  |  |
| High school | 37 (29.65) | 41 (29.35) |  |  |
| Above high school | 57 (43.84) | 55 (44.53) |  |  |
| Physical activity, MET·min/week, Mean (SE) | 231.42 (54.74) | 174.74 (22.11) | t=0.84 | 0.400 |
| Physical activity, MET·min/week, n (%) |  |  | χ^2^=2.030 | 0.154 |
| <450 | 122 (84.12) | 125 (90.09) |  |  |
| ≥450 | 20 (15.88) | 17 (9.91) |  |  |
| Marital status, n (%) |  |  | χ^2^=4.081 | 0.130 |
| Single | 5 (2.00) | 7 (6.12) |  |  |
| Married | 62 (45.75) | 61 (50.52) |  |  |
| Unknown | 75 (52.25) | 74 (43.36) |  |  |
| Dyslipidemia, n (%) |  |  | χ^2^=0.564 | 0.453 |
| No | 11 (5.74) | 5 (3.61) |  |  |
| Yes | 131 (94.26) | 137 (96.39) |  |  |
| Hypertension, n (%) |  |  | χ^2^=0.272 | 0.602 |
| No | 6 (1.73) | 4 (2.55) |  |  |
| Yes | 136 (98.27) | 138 (97.45) |  |  |
| DM, n (%) |  |  | χ^2^=0.179 | 0.672 |
| No | 69 (48.86) | 63 (51.90) |  |  |
| Yes | 73 (51.14) | 79 (48.10) |  |  |
| Cancer, n (%) |  |  | χ^2^=2.289 | 0.130 |
| No | 105 (66.82) | 111 (74.98) |  |  |
| Yes | 37 (33.18) | 31 (25.02) |  |  |
| Stroke, n (%) |  |  | χ^2^=0.491 | 0.483 |
| No | 111 (79.53) | 107 (75.60) |  |  |
| Yes | 31 (20.47) | 35 (24.40) |  |  |
| CHD, n (%) |  |  | χ^2^=2.088 | 0.148 |
| No | 83 (56.87) | 68 (46.70) |  |  |
| Yes | 59 (43.13) | 74 (53.30) |  |  |
| Antipsychotics, n (%) |  |  | χ^2^=0.002 | 0.964 |
| No | 139 (98.97) | 139 (98.93) |  |  |
| Yes | 3 (1.03) | 3 (1.07) |  |  |
| Aldosterone receptor antagonists, n (%) |  |  | χ^2^=0.380 | 0.538 |
| No | 126 (88.04) | 128 (90.78) |  |  |
| Yes | 16 (11.96) | 14 (9.22) |  |  |
| ACEI, n (%) |  |  | χ^2^=1.529 | 0.216 |
| No | 139 (97.58) | 135 (92.70) |  |  |
| Yes | 3 (2.42) | 7 (7.30) |  |  |
| Course of HF, Mean (SE) | 9.91 (0.87) | 10.99 (0.77) | t=-0.92 | 0.358 |
| BMI, kg/m^2^, Mean (SE) | 31.41 (0.68) | 32.31 (0.57) | t=-0.86 | 0.392 |
| BMI, n (%) |  |  | χ^2^=1.721 | 0.423 |
| <25 | 29 (19.99) | 18 (13.95) |  |  |
| [25, 30) | 35 (24.94) | 43 (26.65) |  |  |
| ≥30 | 78 (55.07) | 81 (59.40) |  |  |
| Vitamin D | 34.27 (1.48) | 34.51 (1.28) | t=-0.11 | 0.910 |
| Total energy intake, Mean (SE) | 1666.03 (91.13) | 1714.15 (67.23) | t=-0.40 | 0.690 |
| HEI-2015, Mean (SE) | 53.11 (1.48) | 49.63 (1.37) | t=1.55 | 0.124 |
| Mg, mg, Mean (SE) | 248.72 (11.98) | 231.98 (8.27) | t=1.04 | 0.302 |
| Ca, mg, Mean (SE) | 821.89 (43.29) | 774.51 (41.82) | t=0.69 | 0.493 |
| Alcohol, gm, Mean (SE) | 6.12 (1.95) | 3.48 (1.01) | t=1.15 | 0.251 |
| MDS, n (%) |  |  | χ^2^=0.720 | 0.396 |
| ≤2 | 77 (49.52) | 67 (43.47) |  |  |
| >2 | 65 (50.48) | 75 (56.53) |  |  |
| Smoking status, n (%) |  |  | χ^2^=7.080 | **0.029** |
| No smoking | 74 (57.13) | 61 (38.76) |  |  |
| Quit smoking | 52 (35.06) | 64 (51.29) |  |  |
| Currently smoking | 16 (7.81) | 17 (9.96) |  |  |
| Cotinine, ng/mL, n (%) |  |  | χ^2^=6.653 | **0.036** |
| <0.05 | 81 (65.59) | 69 (53.19) |  |  |
| [0.05, 3) | 38 (23.96) | 45 (25.90) |  |  |
| ≥3 | 23 (10.45) | 28 (20.91) |  |  |

t: t test, χ^2^: chi-square test.

HF: heart failure, SE: standard error, PIR: poverty income ratio, DM: diabetes mellitus, CHD: coronary heart disease, ACEI: angiotensin-converting enzyme inhibitors, BMI: body mass index, HEI-2015: the Healthy Eating Index-2015, Mg: magnesium, Ca: calcium, eGFR: estimated glomerular filtration rate, PPI: proton pump inhibitor, MDS: magnesium depletion score.

**Table S7. Potential regulating effect of MDS on association between tobacco exposure and depression after PSM**

| Variables | OR (95% CI) | *P* |
| --- | --- | --- |
| MDS: ≤2 |  |  |
| Smoking status |  |  |
| No smoking | Ref |  |
| Quit smoking | 1.64 (0.74-3.64) | 0.209 |
| Currently smoking | 1.23 (0.44-3.46) | 0.676 |
| MDS: >2 |  |  |
| Smoking status |  |  |
| No smoking | Ref |  |
| Quit smoking | 2.73 (1.38-5.39) | **0.006** |
| Currently smoking | 5.99 (2.83-12.68) | **<0.001** |
| MDS: ≤2 |  |  |
| Cotinine |  |  |
| <0.05 | Ref |  |
| [0.05, 3) | 1.25 (0.60-2.58) | 0.533 |
| ≥3 | 1.60 (0.56-4.60) | 0.368 |
| MDS: >2 |  |  |
| Cotinine |  |  |
| <0.05 | Ref |  |
| [0.05, 3) | 1.38 (0.68-2.83) | 0.356 |
| ≥3 | 5.01 (1.88-13.32) | **0.002** |

MDS: magnesium depletion score, PSM: propensity score matching, OR: odds ratio, CI: confidence interval, Ref: reference.

**Table S8. Characteristics of participants in non-depression group and depression group (leaving out antidepressant use)**

| Variables | Total (n=554) | Depression | | Statistics | *P* |
| --- | --- | --- | --- | --- | --- |
|  |  | No (n=476) | Yes (n=78) |  |  |
| Age, years, Mean (SE) | 72.82 (0.33) | 73.11 (0.37) | 70.95 (0.85) | t=2.17 | **0.033** |
| Gender, n (%) |  |  |  | χ^2^=2.817 | 0.093 |
| Male | 303 (50.81) | 271 (52.69) | 32 (38.52) |  |  |
| Female | 251 (49.19) | 205 (47.31) | 46 (61.48) |  |  |
| Race, n (%) |  |  |  | χ^2^=6.114 | **0.047** |
| White | 321 (79.96) | 287 (81.38) | 34 (70.68) |  |  |
| Black | 116 (9.93) | 100 (9.61) | 16 (12.00) |  |  |
| Others | 117 (10.12) | 89 (9.01) | 28 (17.33) |  |  |
| PIR | 2.47 (0.08) | 2.53 (0.08) | 2.06 (0.30) | t=1.48 | 0.143 |
| PIR, n (%) |  |  |  | χ^2^=7.331 | **0.007** |
| <1.3 | 182 (23.47) | 139 (21.15) | 43 (38.65) |  |  |
| ≥1.3 | 372 (76.53) | 337 (78.85) | 35 (61.35) |  |  |
| Educational level, n (%) |  |  |  | χ^2^=0.116 | 0.944 |
| Under high school | 160 (22.52) | 133 (22.23) | 27 (24.42) |  |  |
| High school | 161 (30.70) | 139 (30.84) | 22 (29.79) |  |  |
| Above high school | 233 (46.78) | 204 (46.93) | 29 (45.79) |  |  |
| Physical activity, MET·min/week, Mean (SE) | 412.96 (40.16) | 445.59 (47.84) | 199.77 (46.61) | t=3.28 | **0.001** |
| Physical activity, MET·min/week, n (%) |  |  |  | χ^2^=2.991 | 0.084 |
| <450 | 430 (77.43) | 360 (75.78) | 70 (88.21) |  |  |
| ≥450 | 124 (22.57) | 116 (24.22) | 8 (11.79) |  |  |
| Marital status, n (%) |  |  |  | χ^2^=9.500 | **0.009** |
| Single | 25 (3.71) | 19 (2.46) | 6 (11.83) |  |  |
| Married | 267 (54.18) | 235 (54.36) | 32 (52.98) |  |  |
| Unknown | 262 (42.11) | 222 (43.17) | 40 (35.19) |  |  |
| Dyslipidemia, n (%) |  |  |  | χ^2^=0.799 | 0.371 |
| No | 45 (7.13) | 41 (7.52) | 4 (4.55) |  |  |
| Yes | 509 (92.87) | 435 (92.48) | 74 (95.45) |  |  |
| Hypertension, n (%) |  |  |  | χ^2^=5.645 | **0.018** |
| No | 16 (2.03) | 11 (1.44) | 5 (5.92) |  |  |
| Yes | 538 (97.97) | 465 (98.56) | 73 (94.08) |  |  |
| DM, n (%) |  |  |  | χ^2^=0.111 | 0.739 |
| No | 273 (53.46) | 234 (53.90) | 39 (50.60) |  |  |
| Yes | 281 (46.54) | 242 (46.10) | 39 (49.40) |  |  |
| Cancer, n (%) |  |  |  | χ^2^=0.083 | 0.773 |
| No | 422 (70.49) | 364 (70.78) | 58 (68.56) |  |  |
| Yes | 132 (29.51) | 112 (29.22) | 20 (31.44) |  |  |
| Stroke, n (%) |  |  |  | χ^2^=4.234 | **0.040** |
| No | 447 (80.80) | 389 (82.54) | 58 (69.41) |  |  |
| Yes | 107 (19.20) | 87 (17.46) | 20 (30.59) |  |  |
| CHD, n (%) |  |  |  | χ^2^=5.738 | **0.017** |
| No | 314 (56.38) | 276 (59.00) | 38 (39.21) |  |  |
| Yes | 240 (43.62) | 200 (41.00) | 40 (60.79) |  |  |
| Antipsychotics, n (%) |  |  |  | χ^2^=0.460 | 0.498 |
| No | 545 (98.90) | 469 (99.02) | 76 (98.15) |  |  |
| Yes | 9 (1.10) | 7 (0.98) | 2 (1.85) |  |  |
| Aldosterone receptor antagonists, n (%) |  |  |  | χ^2^=0.007 | 0.934 |
| No | 495 (89.98) | 425 (90.04) | 70 (89.59) |  |  |
| Yes | 59 (10.02) | 51 (9.96) | 8 (10.41) |  |  |
| ACEI, n (%) |  |  |  | χ^2^=2.764 | 0.096 |
| No | 534 (95.60) | 460 (96.66) | 74 (88.62) |  |  |
| Yes | 20 (4.40) | 16 (3.34) | 4 (11.38) |  |  |
| Course of HF, Mean (SE) | 10.28 (0.50) | 10.22 (0.53) | 10.73 (1.27) | t=-0.37 | 0.713 |
| BMI, kg/m^2^, Mean (SE) | 31.57 (0.35) | 31.53 (0.36) | 31.86 (1.01) | t=-0.31 | 0.755 |
| BMI, n (%) |  |  |  | χ^2^=4.378 | 0.112 |
| <25 | 92 (15.57) | 79 (14.72) | 13 (21.15) |  |  |
| [25, 30) | 170 (30.01) | 152 (31.82) | 18 (18.15) |  |  |
| ≥30 | 292 (54.42) | 245 (53.46) | 47 (60.70) |  |  |
| Vitamin D | 35.72 (0.77) | 35.53 (0.84) | 36.96 (2.06) | t=-0.60 | 0.549 |
| Total energy intake, Mean (SE) | 1757.48 (40.45) | 1758.65 (40.90) | 1749.86 (114.88) | t=0.07 | 0.941 |
| HEI-2015, Mean (SE) | 52.66 (0.77) | 53.01 (0.84) | 50.36 (1.72) | t=1.37 | 0.172 |
| Mg, mg, Mean (SE) | 252.37 (5.76) | 253.36 (6.25) | 245.91 (14.51) | t=0.46 | 0.647 |
| Ca, mg, Mean (SE) | 805.01 (24.30) | 812.82 (25.52) | 753.99 (59.90) | t=0.92 | 0.359 |
| Alcohol, gm, Mean (SE) | 6.74 (0.84) | 7.09 (0.90) | 4.39 (1.99) | t=1.23 | 0.223 |
| eGFR, Mean (SE) | 66.00 (1.12) | 65.24 (1.06) | 70.97 (3.27) | t=-1.78 | 0.078 |
| Score of eGFR, n (%) |  |  |  | χ^2^=1.628 | 0.443 |
| 0 | 67 (11.60) | 54 (11.00) | 13 (15.49) |  |  |
| 1 | 267 (48.40) | 225 (47.51) | 42 (54.17) |  |  |
| 2 | 220 (40.00) | 197 (41.48) | 23 (30.34) |  |  |
| Diuretics, n (%) |  |  |  | χ^2^=0.238 | 0.626 |
| No | 220 (36.96) | 180 (36.48) | 40 (40.09) |  |  |
| Yes | 334 (63.04) | 296 (63.52) | 38 (59.91) |  |  |
| PPI, n (%) |  |  |  | χ^2^=1.021 | 0.312 |
| No | 398 (69.40) | 342 (68.54) | 56 (75.03) |  |  |
| Yes | 156 (30.60) | 134 (31.46) | 22 (24.97) |  |  |
| Score of drinking, n (%) |  |  |  | χ^2^=0.000 | 0.985 |
| 0 | 474 (84.79) | 414 (84.77) | 60 (84.87) |  |  |
| 1 | 80 (15.21) | 62 (15.23) | 18 (15.13) |  |  |
| MDS, n (%) |  |  |  | χ^2^=0.521 | 0.470 |
| ≤2 | 311 (54.34) | 263 (53.52) | 48 (59.68) |  |  |
| >2 | 243 (45.66) | 213 (46.48) | 30 (40.32) |  |  |
| Score of MDS, n (%) |  |  |  | χ^2^=7.272 | 0.201 |
| 0 | 20 (2.12) | 14 (1.89) | 6 (3.62) |  |  |
| 1 | 107 (17.02) | 89 (15.76) | 18 (25.24) |  |  |
| 2 | 184 (35.19) | 160 (35.86) | 24 (30.82) |  |  |
| 3 | 173 (32.97) | 152 (32.84) | 21 (33.81) |  |  |
| 4 | 67 (12.52) | 59 (13.52) | 8 (5.98) |  |  |
| 5 | 3 (0.18) | 2 (0.12) | 1 (0.52) |  |  |
| Smoking status, n (%) |  |  |  | χ^2^=3.230 | 0.199 |
| No smoking | 235 (43.41) | 206 (45.16) | 29 (31.97) |  |  |
| Quit smoking | 241 (45.54) | 204 (44.16) | 37 (54.55) |  |  |
| Currently smoking | 78 (11.05) | 66 (10.68) | 12 (13.48) |  |  |
| Cotinine, ng/mL, Mean (SE) | 40.59 (5.23) | 42.22 (5.81) | 29.93 (9.15) | t=1.14 | 0.255 |
| Cotinine, ng/mL, n (%) |  |  |  | χ^2^=1.109 | 0.574 |
| <0.05 | 292 (58.96) | 260 (60.03) | 32 (51.97) |  |  |
| [0.05, 3) | 148 (23.21) | 120 (22.64) | 28 (26.98) |  |  |
| ≥3 | 114 (17.82) | 96 (17.33) | 18 (21.05) |  |  |

t: t test, χ^2^: chi-square test.

HF: heart failure, SE: standard error, PIR: poverty income ratio, DM: diabetes mellitus, CHD: coronary heart disease, ACEI: angiotensin-converting enzyme inhibitors, BMI: body mass index, HEI-2015: the Healthy Eating Index-2015, Mg: magnesium, Ca: calcium, eGFR: estimated glomerular filtration rate, PPI: proton pump inhibitor, MDS: magnesium depletion score.

**Table S9. Covariates associated with depression (leaving out antidepressant use)**

| Variables | OR (95%CI) | P |
| --- | --- | --- |
| Age | 0.95 (0.91-0.99) | **0.028** |
| Gender |  |  |
| Male | Ref |  |
| Female | 1.78 (0.88-3.58) | 0.106 |
| Race |  |  |
| White | Ref |  |
| Black | 1.44 (0.71-2.91) | 0.309 |
| Others | 2.21 (1.06-4.64) | **0.036** |
| PIR | 0.77 (0.51-1.16) | 0.211 |
| PIR |  |  |
| <1.3 | Ref |  |
| ≥1.3 | 0.43 (0.22-0.83) | **0.012** |
| Educational level |  |  |
| Under high school | Ref |  |
| High school | 0.88 (0.41-1.90) | 0.741 |
| Above high school | 0.89 (0.39-2.03) | 0.775 |
| Physical activity | 0.99 (0.99-0.99) | **0.025** |
| Physical activity level |  |  |
| <450 | Ref |  |
| ≥450 | 0.42 (0.15-1.17) | 0.094 |
| Marital status |  |  |
| Single | Ref |  |
| Married | 0.20 (0.04-0.96) | **0.045** |
| Unknown | 0.17 (0.04-0.79) | **0.024** |
| Dyslipidemia |  |  |
| No | Ref |  |
| Yes | 1.71 (0.51-5.66) | 0.377 |
| Hypertension |  |  |
| No | Ref |  |
| Yes | 0.23 (0.06-0.89) | **0.033** |
| DM |  |  |
| No | Ref |  |
| Yes | 1.14 (0.52-2.52) | 0.741 |
| Cancer |  |  |
| No | Ref |  |
| Yes | 1.11 (0.54-2.30) | 0.774 |
| Stroke |  |  |
| No | Ref |  |
| Yes | 2.08 (1.00-4.35) | 0.050 |
| CHD |  |  |
| No | Ref |  |
| Yes | 2.23 (1.12-4.44) | **0.023** |
| Antipsychotics |  |  |
| No | Ref |  |
| Yes | 1.89 (0.28-12.72) | 0.507 |
| Aldosterone receptor antagonists |  |  |
| No | Ref |  |
| Yes | 1.05 (0.32-3.44) | 0.934 |
| ACEI |  |  |
| No | Ref |  |
| Yes | 3.72 (0.68-20.41) | 0.129 |
| Course of HF | 1.01 (0.98-1.03) | 0.694 |
| BMI | 1.01 (0.97-1.05) | 0.747 |
| BMI |  |  |
| <25 | Ref |  |
| [25, 30) | 0.40 (0.15-1.03) | 0.057 |
| ≥30 | 0.79 (0.35-1.80) | 0.571 |
| Vitamin D | 1.00 (0.99-1.02) | 0.528 |
| Total energy intake | 1.00 (1.00-1.00) | 0.941 |
| HEI-2015 | 0.99 (0.96-1.01) | 0.179 |
| Mg | 1.00 (1.00-1.00) | 0.646 |
| Ca | 1.00 (1.00-1.00) | 0.394 |
| Alcohol | 0.99 (0.97-1.01) | 0.405 |
| Antidepressants |  |  |
| No | Ref |  |
| Yes | 2.89 (1.60-5.20) | **<0.001** |

OR: odds ratio, CI: confidence interval, Ref: reference, PIR: poverty income ratio, DM: diabetes mellitus, CHD: coronary heart disease, ACEI: angiotensin-converting enzyme inhibitors, HF: heart failure, BMI: body mass index, HEI-2015: the Healthy Eating Index-2015, Mg: magnesium, Ca: calcium.
